# Evidence for direct and sleep‐moderated relationships between aquaporin‐4 genetic variants and Alzheimer's disease phenotypes

**DOI:** 10.1002/alz.71516

**Published:** 2026-05-29

**Authors:** Tenielle Porter, Ayeisha Milligan Armstrong, Eleanor K. O'Brien, Vincent Doré, Pierrick Bourgeat, Mitchell Turner, Paul Maruff, Christopher C. Rowe, Belinda M. Brown, Victor L. Villemagne, Stephanie R. Rainey‐Smith, Simon M. Laws, AIBL Research Group

**Affiliations:** ^1^ Centre for Precision Health Edith Cowan University Joondalup Western Australia Australia; ^2^ Collaborative Genomics and Translation Group, School of Medical and Health Sciences Edith Cowan University Joondalup Western Australia Australia; ^3^ Curtin Medical School Curtin University Bentley Western Australia Australia; ^4^ Australian E‐Health Research Centre, CSIRO Parkville Victoria Australia; ^5^ Department of Molecular Imaging and Therapy Austin Health Heidelberg Victoria Australia; ^6^ Australian E‐Health Research Centre, CSIRO Herston Queensland Australia; ^7^ School of Medical and Health Sciences Edith Cowan University Joondalup Western Australia Australia; ^8^ Florey Institute of Neuroscience and Mental Health The University of Melbourne Parkville Victoria Australia; ^9^ Cogstate Ltd Melbourne Victoria Australia; ^10^ Centre for Healthy Ageing, Health Futures Institute, Murdoch University Murdoch Western Australia Australia; ^11^ Department of Psychiatry University of Pittsburgh Pittsburgh Pennsylvania USA; ^12^ School of Psychological Science University of Western Australia Crawley Western Australia Australia

**Keywords:** Amyloid beta, aquaporin‐4 (AQP4), brain volume, cognition, glymphatic system, sleep

## Abstract

**INTRODUCTION:**

Variants in the aquaporin‐4 gene (*AQP4*) have been associated with Alzheimer's disease (AD) diagnosis, cognition, and brain amyloid beta (Aβ) and may affect the sleep and Aβ relationship. Their association with other AD‐related phenotypes/disease progression remain largely unknown.

**METHODS:**

Associations between *AQP4* variants, self‐reported sleep measures, and AD‐related phenotypes in cognitively unimpaired individuals with evidence of Aβ accumulation were examined using data from the Australian Imaging, Biomarkers and Lifestyle study.

**RESULTS:**

*AQP4* variants were directly associated with regional brain volumes, atrophy, and cognition. They were also associated with differences in regional brain volumes and atrophy in interaction with sleep duration, latency, and quality. Finally, *AQP4* variants were associated with cognitive decline in interaction with sleep disturbances.

**DISCUSSION:**

These findings support a relationship between *AQP4* and AD phenotypes, both directly and through their interaction with sleep.

## Highlights

- This was multimodal study of at‐risk individuals integrating genetics, neuroimaging, cognition, and sleep.
- *AQP4* genetic variants were associated with cognition, both directly and through interactions with sleep quality.
- Sleep duration, latency onset, and global quality consistently moderated the associations between *AQP4* genetic variants and cognition and brain volumetrics.
- Α broad relationship was identified between *AQP4* genetic variation and AD, with sleep as a potential modifier.

## 1 INTRODUCTION

Alzheimer's disease (AD) is characterized by the accumulation in the brain of the amyloid beta (Aβ) protein and the subsequent deposition of hyperphosphorylated tau protein. These events drive neurotoxicity and brain atrophy, which manifest symptomatically as progressive cognitive decline and ultimately dementia.^1^, ^2^ In sporadic or late‐onset AD, decreased clearance of Aβ from the brain is a hallmark feature.^2^ There is growing evidence that Aβ is cleared from the brain via a glymphatic clearance system, which is partially mediated by astrocytic endfeet.^3^, ^4^ A key component of this system is the movement of interstitial fluid along perivascular pathways, a process facilitated by the aquaporin‐4 (AQP4) protein water channel,^5^ which is primarily located at the perivascular endfeet of astrocytic processes. It is thought that AQP4 impacts Aβ clearance by regulating these fluid dynamics. AQP4 is also proposed to play a key role in mediating other astrocytic functions such as glutamate, potassium, and calcium regulation^6^ and neuroinflammation.^7^

AQP4 localization changes with aging and the occurrence of AD. In *post mortem* brain tissue, AQP4 is uniformly expressed in young brains; however, with aging, localization shifts away from the perivascular space, accompanied by increasing *AQP4* gene expression.^8^ In AD, loss of perivascular AQP4 localization is substantial compared to healthy age‐matched controls. This loss is associated with worsening AD pathology, including increased Aβ plaques and tau accumulation, supporting a critical role for AQP4 in mediating perivascular function.^8^ These findings in humans are supported by transgenic mouse models, which show that *AQP4* gene deletion results in increased brain Aβ plaque formation, greater cognitive impairment, loss of synapse‐related proteins, astrogliosis, and increased oxidative stress within the brain (as reviewed elsewhere^9^).

Given that AQP4 has been identified as an important mediator of brain Aβ clearance, variation within the *AQP4* gene has been investigated in relation to neurodegenerative diseases and their associated phenotypes.^10^, ^11^, ^12^, ^13^ Previous studies showed that genetic variants within *AQP4* were associated with differences in the rate of cognitive decline in adults with mild cognitive impairment (MCI),^11^ as well as in individuals diagnosed with AD^10^ or Parkinson's disease.^12^ Further, associations between *AQP4* genetic variants with clinical disease progression from MCI to AD and increasing brain Aβ burden have been shown.^11^

**RESEARCH IN CONTEXT**

1. **Systematic review**: A review of prior studies, identified through sources such as PubMed, suggests that the *AQP4* gene is associated with Aβ burden and cognition. Genetic studies further suggest associations between *AQP4*, disease progression, and sleep interactions. However, findings have been inconsistent, and the underlying mechanisms remain unclear.
2. **Interpretation**: These results strengthen the evidence that *AQP4* genetic variation is associated with AD‐related phenotypes, including cognition and brain structure, in individuals at risk for AD, with sleep quality acting as a potential modifier.
3. **Future directions**: Future work should replicate these findings in independent and more diverse cohorts, investigate functional mechanisms of implicated variants, and test whether sleep interventions can modify risk in genetically susceptible individuals.

A bi‐directional relationship has been observed between suboptimal sleep and increasing brain Aβ accumulation.^14^, ^15^, ^16^ This relationship, in part, is believed to result from reduced clearance via the glymphatic system,^3^ which is proposed to function primarily during sleep.^4^ Studies in mice^17^, ^18^ and humans^16^, ^19^, ^20^ have shown that while suboptimal sleep is associated with an increased brain Aβ burden or accumulation, the opposite has been observed with good‐quality sleep. The underlying mechanism of this association has been suggested to be through enhanced clearance. Importantly, a previous study utilizing data from the Australian Imaging, Biomarker and Lifestyle (AIBL) cohort reported that the relationship between sleep and cross‐sectional brain Aβ burden was moderated by genetic variants in *AQP4*.^13^ Given that poor sleep is reported widely in adults living with AD,^21^, ^22^, ^23^ a greater understanding of how sleep may be related to pathological hallmarks of AD, as well as related phenotypes such as cognitive impairment and changes in brain volume measures, is required. In conjunction with this, understanding how genetic variation and potentially modifiable factors, such as sleep, interact is also of interest.

The *AQP4* gene contains many common genetic variants, known as single‐nucleotide polymorphisms (SNPs), which can influence AQP4 protein structure, function, or regulation. Given previous findings from the AIBL study showing associations with Aβ burden in the cross‐sectional setting discussed above,^13^ it was hypothesized that *AQP4* SNPs and sleep measures may be associated with other AD‐related phenotypes. The current study addressed this by assessing relationships between *AQP4* SNPs, their interactions with self‐reported sleep measures, and a greater range of AD‐related phenotypes (brain Aβ burden, brain volumetrics, and cognitive measures) in both the cross‐sectional and longitudinal setting. In total, 13 SNPs were selected to capture genetic variation across the gene. Given the relationship between poor sleep and Aβ accumulation described above, in conjunction with sleep disturbances being observed in preclinical AD cohorts, the current study focused on cognitively unimpaired (CU) older adults at an increased risk of AD due to brain Aβ accumulation.

## 2 METHODS

### 2.1 Study participants

This study utilized data from participants of the Australian Imaging, Biomarkers and Lifestyle (AIBL) study cohort, whose inclusion and exclusion criteria, as well as protocols, have been described in detail elsewhere.^24^, ^25^ For the current study, inclusion was limited to those who had genetic, sleep measure, and covariate data available. Further, this sample was limited to CU participants at increased risk of AD due to Aβ accumulation. Specifically, classification as an Aβ accumulator was defined by the individual having either (i) a baseline Centiloid score of >20 or (ii) a positive slope of Aβ accumulation over at least three assessments (minimum of 36 months). This resulted in a total of 351 participants being identified as CU Aβ accumulators with genetic, sleep, and covariate data available. Sample sizes for specific analyses subsequently varied based on the availability of data for the outcome variable. Finally, to be included in longitudinal analysis, a minimum of three time points of data for the outcome variable of interest was required. The AIBL study is approved by the human research ethics committees at Austin Health, St Vincent's Health, Ramsay Health Care WA|SA, and Edith Cowan University. Participants provided written informed consent prior to undergoing study procedures.

### 2.2 Sleep measures

The Pittsburgh Sleep Quality Index (PSQI) was used to assess self‐reported sleep quality over a 1‐month interval. The PSQI is a 19‐item questionnaire used to calculate seven sleep components, which are subsequently summed to derive a global PSQI score.^26^ A higher global PSQI score is indicative of worse overall sleep quality. For the current study, the following sleep components were used: global PSQI, sleep duration (in hours), sleep onset latency (in minutes), sleep disturbances (in quantity), and sleep efficiency (as a percentage). Individual PSQI sleep components and the global score were analyzed as continuous variables.

### 2.3 Brain imaging

Positron emission tomography (PET) imaging was used to quantify cortical Aβ levels. One of five Aβ tracers was used: ^11^C‐Pittsburgh Compound B, ^18^F‐Flutemetamol, ^18^F‐Florbetapir, ^18^F‐Florbetaben, or ^18^F‐NAV4694. CapAIBL was employed to analyze images by generating tracer‐specific standardized uptake value ratios (SUVRs), which were then transformed into Centiloids, as previously described.^27^, ^28^

Brain volumes were quantified from magnetic resonance imaging (MRI) scans at T1 using the magnetization‐prepared rapid gradient echo (MPRAGE) protocol. Estimation of cortical brain volumes (gray matter volume, white matter volume, hippocampal volume, and ventricular volume) was performed using CurAIBL, as previously described.^29^ Brain volumes underwent correction for intracranial volumes and the MRI scanner used.

### 2.4 Cognition

AIBL protocols for cognitive testing have been described in detail elsewhere.^24^, ^25^, ^30^, ^31^ Participants underwent comprehensive neuropsychological assessments using cognitive test batteries that measure six cognitive domains known to be impacted in AD: executive function, episodic recall, recognition memory, language performance, attention and processing speed, and global cognition assessed via the AIBL Preclinical Alzheimer's Cognitive Composite (AIBL PACC). The component tests from which these cognitive domain scores are derived are listed in Table S1.

### 2.5 Genotyping and SNP selection

Participants’ DNA was extracted from whole blood using QIAamp DNA blood spin column kits (Qiagen, Valencia, CA, USA) in accordance with the manufacturer's instructions, as previously described.^24^, ^25^ Genotyping of apolipoprotein E (*APOE*), a major genetic risk factor for AD, was completed as described previously^32^, ^33^ using TaqMan genotyping assays (Life Technologies, USA) on a QuantStudio 12k Flex Real‐Time PCR system (Applied Biosystems, USA). SNP genotype data were obtained using the Axiom Precision Medicine Diversity Array (Applied Biosystems). The TOPMed Imputation Server was used to impute all genotype data against the TOPMed panel.^34^, ^35^ *AQP4* SNPs were selected based on a quality‐control and fine‐mapping approach, as previously described in detail.^13^ Briefly, this included removal of SNPs with low call rates (< 95%), a low minor allele frequency (< 0.05), or deviation from Hardy Weinberg Equilibrium, followed by a linkage disequilibrium pruning step. This resulted in 13 SNPs selected for analysis, providing full coverage of the *AQP4* gene. Eight of these variants have high RegulomeDB and ForgeDB scores, meaning they are predicted to have regulatory functions (Table S2). For a visual representation of their location in relation to the *AQP4* gene region see Figure S1.

### 2.6 Statistical analysis

All statistical analyses were conducted using R (version 4.3.2) in RStudio (2023.09.1+494) for MacOS. Demographic summary statistics were compiled for the included 351 participants as a whole group, as well as for the subsets of participants included in different outcome variable analyses.

Sleep component data were collected at multiple time points throughout the study. However, when investigating these measures, scores from the first time point, when all required data were collected, were used. As longitudinal changes in AD‐related phenotypes were investigated, the stability of sleep measures over time was assessed using linear mixed‐effects models and Spearman correlation analyses. No significant effect of time on sleep measures was observed, and moderate to strong within‐individual correlations were identified across time points, supporting the relative stability of sleep measures over follow‐up (Tables S3 and S4).

When assessing AD‐related phenotypes, data collected at the same time point as the first sleep assessment were examined in the cross‐sectional analyses. For longitudinal analyses of AD‐related phenotypes, participants’ rates of change in each phenotype were first calculated, and these values were subsequently assessed as outlined below. Longitudinal changes in brain Aβ (Centiloid) were calculated using a least‐squares linear regression fitted to the three or more data points available for each participant, where the resulting slope was considered the participant's rate of Aβ accumulation.^36^ For brain volumes and cognitive domains, linear mixed models were implemented using the lmer package to calculate individual intercepts and slopes for each phenotype.

The 13 *AQP4* SNPs were investigated under dominant (major allele homozygotes vs minor allele carriers) and recessive (major allele carriers vs minor allele homozygotes) genetic models for each analysis. Here, the minor allele refers to the allele that is less abundant in Caucasian populations. All direct associations were assessed via linear regression using the *“*lm*”* function, with age, sex, and *APOE* ε4 carrier status (binary; presence/absence) were included as a covariate. If longitudinal phenotypes were being investigated, then baseline (Centiloid) or intercept (brain volume/cognition) value was included as a covariate in the model. The models are defined as follows:

$$\begin{array}{c} \text{Brain}\;A\beta /\text{Brain}\;\text{volume}/\text{Cognition}\;\text{measure}\approx \;\\ \text{AQP}4\text{SNP}+\text{Age}+\text{Sex}+\text{APOE}\;\varepsilon 4, \end{array}$$

$$\begin{array}{c} \text{Brain}\;A\beta /\text{Brain}\;\text{volume}/\text{Cognition}\;\text{slope}\;\text{measure}\approx \text{AQP}4\;\text{SNP}\\ +\text{Baseline}\;\text{age}+\text{Baseline}\;/\text{Intercept}\;\text{AD}-\text{related}\;\text{measure}\\ +\text{Sex}+\text{APOE}\;\varepsilon 4 \end{array}$$

To determine the relationship of the interaction between genetic variation within *AQP4* and sleep quality measures and AD‐related phenotypes, linear regression models were run using the “lm” function with a *SNP × Sleep measure* interaction term for each combination of SNP, sleep measure, and AD‐related phenotype. Sleep measures were investigated as continuous variables. These models included age, sex, *APOE* ε4 status, Geriatric Depression Scale (GDS) score, body mass index (BMI), and cardiovascular disease (CVD) as covariates.^13^, ^14^, ^16^ The CVD risk score was calculated by summing an individual's medical history (binary; history/no history) of heart disease, hypertension, angina, and stroke. In addition, when the outcome variable was investigated longitudinally, the baseline (Centiloid) or intercept (brain volume/cognition) value was included as a covariate in the models. The models are defined as follows:

$$\begin{array}{c} \text{Brain}\;A\beta /\text{Brain}\;\text{volume}/\text{Cognition}\;\text{measure}\approx \text{AQP}4\;\text{SNP}\times \\ \text{Sleep}\text{measure}+\text{Age}+\text{Sex}+\text{APOE}\;\varepsilon 4+\text{CVD}+\text{GDS}+\text{BMI} \end{array}$$

$$\begin{array}{c} \text{Brain}\;A\beta /\text{Brain}\;\text{volume}\;/\text{Cognition}\;\text{slope}\;\text{measure}∼\text{AQP}4\;\\ \text{SNP}\times \text{Baseline}\text{sleep}\text{measure}+\text{Baseline}\text{age}+\text{Baseline}/\text{intercept}\;\\ \text{AD}-\text{related}\text{measure}+\text{Sex}+\text{APOE}-\varepsilon 4+\text{CVD}+\text{GDS}+\text{BMI} \end{array}$$

The false discovery rate (FDR) method^37^ was used to correct for multiple testing within each outcome variable and analysis type (*AQP4* SNPs or *AQP4* SNPs × Sleep measure), reflecting the hypothesis‐driven research questions (represented as *q* value). Results from dominant and recessive genetic models were jointly included in the same multiple testing correction framework. Effect sizes were calculated by Cohen's *d*.

## 3 RESULTS

### 3.1 Demographic and clinical characteristics

This study included 351 participants at an increased risk of AD, defined as CU at baseline and an Aβ accumulator, with available sleep measures and genetic data. Of these participants, all 351 had Aβ data, 277 had brain volume data, and 328 had cognition data available at the same time point as their sleep data for cross‐sectional analyses. For longitudinal analyses, 317 participants had Aβ data, 108 had brain volume data, and 302 had cognition data. Demographic and clinical information, as well as summary statistics for each group, are presented in Table 1, with extended demographic and phenotype summaries for the sub‐cohorts provided in Table S5. Baseline comparisons between individuals included in longitudinal analyses and those excluded due to data availability showed no consistent differences in demographic characteristics or genotype frequencies (Table S6).

**TABLE 1:** Participant demographics.

		Cross‐sectional data available			Longitudinal data available		
	Genetic and sleep data	Brain Aβ data	Brain MRI data	Cog data	Brain Aβ data	Brain MRI data	Cog data
***n***	351	351	277	328	317	108	302
**Age** *mean (SD)*	74.68 (6.96)	74.68 (6.96)	74.52 (7.11)	74.65 (6.94)	74.31 (6.95)	75.17 (6.24)	74.89 (7.06)
**Sex** *n (%)*							
*Male*	194 (55.3)	194 (55.3)	160 (57.8)	181 (55.2)	177 (55.8)	61 (56.5)	166 (55)
*Female*	157 (44.7)	157 (44.7)	117 (42.2)	147 (44.8)	140 (44.2)	47 (43.5)	136 (45)
***APOE*** *n (%)*							
*ε4−*	235 (67)	235 (67)	182 (65.7)	220 (67.1)	218 (68.8)	73 (67.6)	207 (68.5)
*ε4+*	116 (33)	116 (33)	95 (34.3)	108 (32.9)	99 (31.2)	35 (32.4)	95 (31.5)
**BMI** *mean (SD)*	26.44 (4.27)	26.44 (4.27)	26.4 (4.32)	26.49 (4.29)	26.53 (4.39)	26.78 (4.21)	26.46 (4.24)
**GDS**> *mean (SD)*	1.28 (1.27)	1.28 (1.27)	1.21 (1.21)	1.26 (1.27)	1.28 (1.27)	1.28 (1.29)	1.26 (1.27)
**CVD Risk** *mean (SD)*	0.44 (0.53)	0.44 (0.53)	0.41 (0.51)	0.44 (0.53)	0.44 (0.53)	0.43 (0.48)	0.44 (0.53)

*Note*: Summary statistics for participant demographics at current study baseline. To increase sample sizes for each outcome variable participants were sub‐clustered depending on available outcome data.

Abbreviations: Aβ, amyloid beta; *APOE*, apolipoprotein E; BMI, body mass index; Cog, cognition; CVD, cardiovascular disease; GDS, Geriatric Depression Scale; MRI, magnetic resonance imaging; SD, standard deviation.

### 3.2 AQP4 SNP rs162007 is associated with cognitive performance

**FIGURE 1:**
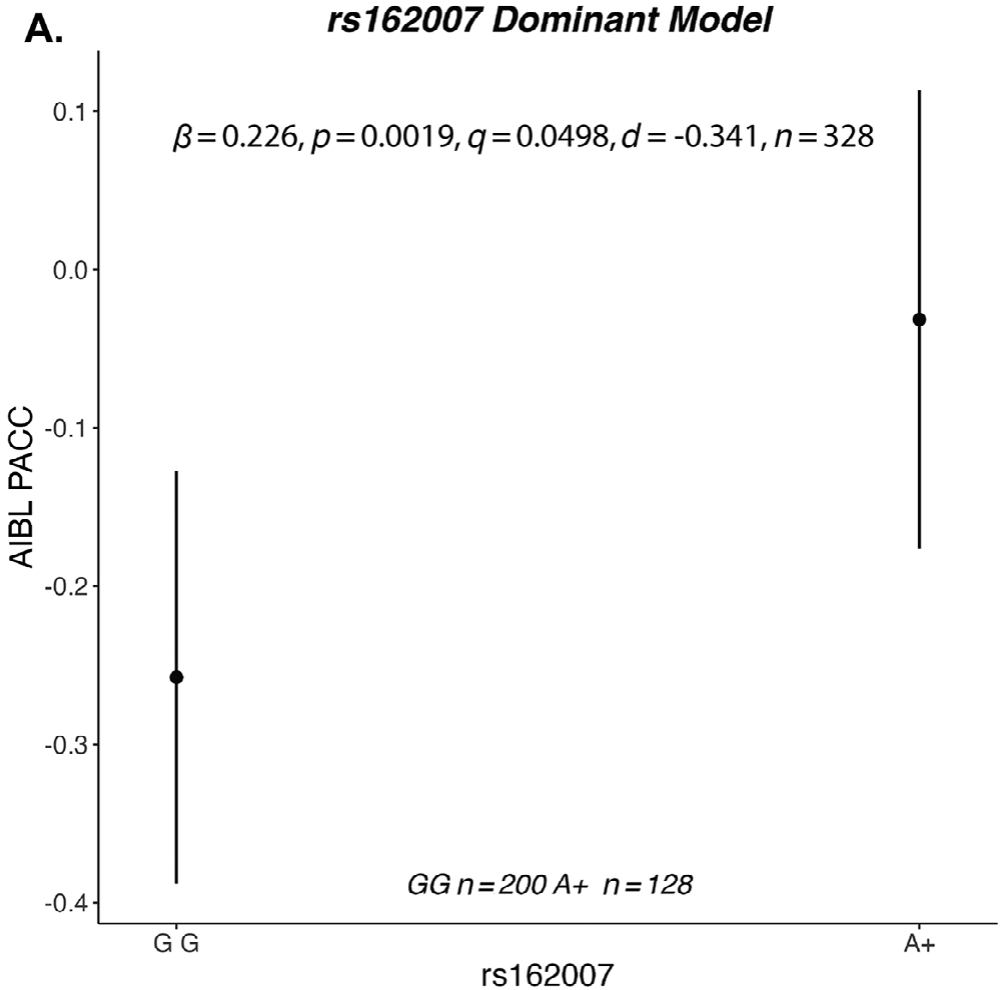
Association between *AQP4* rs162007 and AIBL PACC. Representation of estimated marginal means for (A) rs162007 under a dominant model for cross‐sectional AIBL PACC. The β coefficient represents the effect of the group containing the minor allele (minor allele carriers under dominant genetic model, minor allele homozygotes under recessive genetic model). The *p* value represents the nominal association of the SNP on the AD‐related measure. The *d* value represents Cohen's *d* effect size. Covariates: age, sex, *APOE* ε4, CVD risk score, GDS score, and BMI with the addition of education when examining cognition. AD, Alzheimer's disease; AIBL PACC, Australian Imaging, Biomarkers and Lifestyle study Pre‐clinical Alzheimer's Cognitive Composite; *APOE*, apolipoprotein E; *AQP4*, aquaporin‐4; BMI, body mass index; CVD, cardiovascular disease; FDR, false discovery rate; GDS, Geriatric Depression Scale; SNP, single‐nucleotide polymorphism.

A single FDR significant association of moderate effect size was identified between AQP4 and cognition. Under a dominant genetic model, rs162007 was associated with cross‐sectional differences in AIBL PACC scores, with minor allele carriers (A+) demonstrating better cognitive performance than GG homozygotes (*β *= 0.226 [95% CI: 0.084–0.368], *p *= 0.0019, *q *= 0.0498; *d *= −0.341 [95% CI: −0.556 to −0.127]) (Figure 1). No other direct associations between *AQP4* SNPs and Aβ burden or regional brain volumes survived FDR correction. For full results of direct associations between *AQP4* SNPs (dominant and recessive genetic models) and AD‐related phenotypes see Table S7.

### 3.3 Interactions between *AQP4* genetic variants and sleep quality are associated with differences in brain volume and cognition measures

**FIGURE 2:**
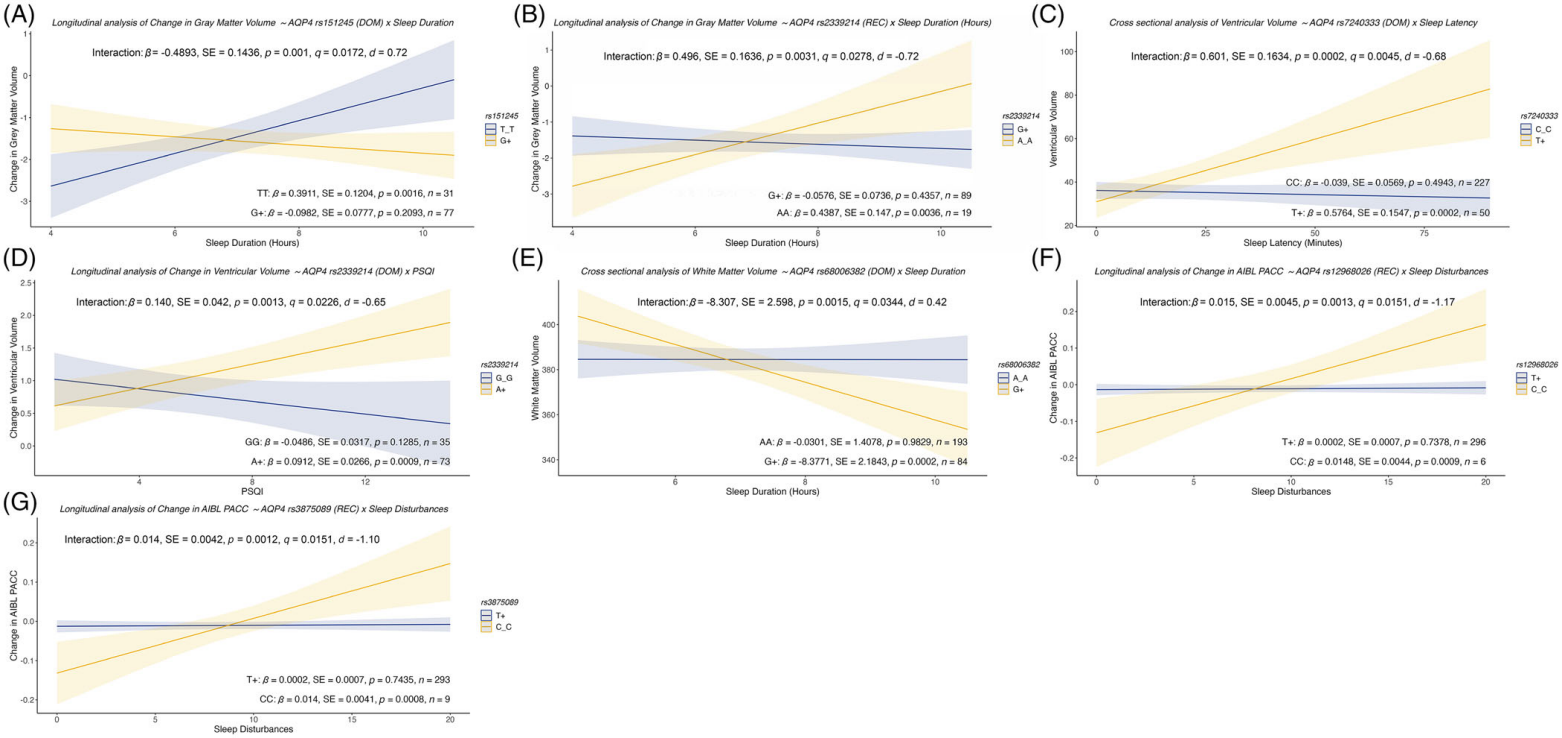
Interaction of *AQP4* SNPs with sleep measures on AD‐related phenotypes. Representation of significant *SNP × Sleep measure* interaction terms with AD‐related phenotypes, surviving FDR correction. For longitudinal changes in gray matter volume, the interaction between (A) rs151245 and sleep duration and (B) rs2339214 and sleep duration. For cross‐sectional ventricular volume measures: interaction between rs7240333 (C) and sleep onset latency. For longitudinal changes in ventricular volume: (D) interaction between rs2339214 and global PSQI score. For cross‐sectional white matter volume measures and interaction between (E) rs68006382 and sleep duration. For measures of longitudinal AIBL PACC: (F) interaction between rs12968026 and sleep disturbances and (G) rs3875089 and sleep disturbances. The main effect of the interaction term between *SNP × Sleep measure* is shown, as is the group effect estimated marginal means for the different genotype groups. The shaded areas represent the 95% confidence intervals surrounding the estimates. The *d* value represents Cohen's *d* effect size. Covariates: age, sex, *APOE* ε4, CVD risk score, GDS score, and BMI with addition of education when examining cognition. AD, Alzheimer's disease; AIBL PACC, Australian Imaging, Biomarkers and Lifestyle study Preclinical Alzheimer's Cognitive Composite; *APOE*, apolipoprotein E; *AQP4*, aquaporin‐4; BMI, body mass index; CVD, cardiovascular disease; DOM/REC, dominant and recessive genetic models; FDR, false discovery rate; GDS, Geriatric Depression Scale; PSQI, Pittsburgh Sleep Quality Index; SNP, single‐nucleotide polymorphism.

Interactions between *AQP4* SNPs and sleep measures, including duration, onset latency, and the global PSQI score, were associated with differences in gray matter, ventricular, and white matter volumes (Table 2; Figure 2). For gray matter volume, interactions between sleep duration and rs151245 or rs2339214 were associated with differences in atrophy, such that the relationship between sleep duration and atrophy varied by genotype, with shorter sleep duration associated with increased atrophy in specific genotype groups. Among individuals homozygous for the rs151245 major allele (TT), each hour less in sleep duration was associated with 0.39 cm^3^/year greater rate of gray matter atrophy (*β *= 0.3911, SE = 0.1204, *p *= 0.0016), which was not seen for G+ carriers (*β *= −0.0982, SE = 0.0777, *p *= 0.2093), with slopes differing significantly (Δ*β *= −0.4893, SE = 0.1436, *p *= 0.001). Each hour less in sleep duration was also associated with a 0.44 cm^3^/year greater rate of gray matter atrophy among rs2339214 homozygous minor allele carriers (AA) (*β *= 0.4387, SE = 0.147, *p *= 0.0036), which differed significantly (Δ*β *= 0.4964, SE = 0.1636, *p = 0.0031*) from G+ carriers (*β *= −0.0576, SE = 0.0736, *p *= 0.4357). For ventricular volume, the interaction between rs7240333 and sleep onset latency was associated with cross‐sectional differences, while the interaction between global PSQI and rs2339214 was associated with[Table alz71516-tbl-0002] longitudinal differences. The rs7240333 minor allele carriers (T+) exhibited 0.57 cm^3^ larger ventricular volumes per minute longer sleep onset latency (*β *= 0.5764, SE = 0.1547, *p *= 0.0002), whereas no association in CC homozygotes was seen (*β *= −0.039, SE = 0.0569, *p *= 0.4943) corresponding to a significant difference in slopes between genotypes (Δ*β *= 0.6153, SE = 0.1634, *p *= 0.0002). Meanwhile, carriers of the rs2339214 minor allele (A+) exhibited 0.09 cm^3^/year greater ventricular expansion per point (worsening) in global PSQI (*β *= 0.0912, SE = 0.0266, *p *= 0.0009), differing significantly (Δ*β *= 0.1397, SE = 0.042, *p *= 0.0013) from the GG homozygotes, where no association was observed (*β *= −0.0486, SE = 0.0317, *p *= 0.1285). Finally, sleep duration interacted with rs68006382 to influence cross‐sectional white matter volume. Carriage of the rs68006382 minor allele (G+) was associated with 8.38 cm^3^ smaller white matter volumes per hour longer sleep duration (*β *= −8.3771, SE = 2.1843, *p *= 0.0002), with no relationship among AA homozygotes (*β *= −0.0301, SE = 1.4078, *p *= 0.9829), reflecting significant slope differences between the genotypes (Δ*β *= −8.3469, SE = 2.5977, *p *= 0.0015).

**TABLE 2 alz71516-tbl-0002:** Significant *AQP4* SNP and sleep measure interaction terms on AD‐related phenotypes.

AD‐related phenotype	Analysis	Sleep measure	SNP	Model	*n*	β [95% CI]	Cohen's D [95% CI]	*p*	*q*
**Brain imaging**									
Gray matter volume	Longitudinal	Duration	rs151245	Dominant	108	−0.489 [−0.774–0.204]	0.721 [0.301–1.141]	0.0010	***0.0172***
			rs2339214	Recessive	108	0.496 [0.172–0.821]	−0.724 [−1.197–0.250]	0.0031	***0.0278***
Ventricular volume	Cross‐sectional	Latency	rs7240333	Dominant	277	0.601 [0.288–0.913]	−0.680 [−1.036–0.325]	0.0002	***0.0045***
	Longitudinal	Global PSQI	rs2339214	Dominant	108	0.140 [0.056–0.223]	−0.649 [−1.036–0.261]	0.0013	***0.0226***
White matter volume	Cross‐sectional	Duration	rs68006382	Dominant	277	−8.307 [−13.404–3.210]	0.424 [0.164–0.683]	0.0015	***0.0344***
**Cognition**									
AIBL PACC	Longitudinal	Disturbances	rs12968026	Recessive	302	0.015 [0.006–0.023]	−1.169 [−1.879–0.460]	0.0013	***0.0151***
			rs3875089	Recessive	302	0.014 [0.005–0.022]	−1.105 [−1.768–0.442]	0.0012	***0.0151***

*Note*: Linear regression results for FDR‐corrected significant associations between AQP4 SNPs × Sleep measure interaction terms on AD‐related phenotypes (for full results table see Table S5). In these models SNPs were run under dominant (major allele homozygote vs minor allele carriers) and recessive (major allele carriers vs minor allele homozygotes) genetic models, and sleep quality measures were included as continuous variables. The β coefficient represents the difference in the slopes between SNP genotypes, the *p* value represents the nominal significance for the SNP × Sleep measure interaction term with the outcome variable of interest, and the *q* value represents the FDR‐corrected *p* values. Covariates: age, sex, APOE ε4, CVD risk score, GDS score, and BMI, with the addition of education when examining cognition.

Abbreviations: AD, Alzheimer's disease; AIBL PACC, Australian Imaging, Biomarkers and Lifestyle study Preclinical Alzheimer's Cognitive Composite; APOE, apolipoprotein E; AQP4, aquaporin‐4; BMI, body mass index; CVD, cardiovascular disease; CI, confidence interval; FDR, false discovery rate; GDS, Geriatric Depression Scale; Model, genetic model (recessive/dominant); PSQI, Pittsburgh Sleep Quality Index; SNP, single‐nucleotide polymorphism.

In addition, interactions between *AQP4* SNPs and sleep disturbances were associated with differences in cognitive decline in the AIBL PACC domain (Table 2; Figure 2). Specifically, rs12968026 and rs3875089 (recessive models) showed interactions with sleep disturbances that were associated with longitudinal differences. For these variants, individuals homozygous for the minor allele exhibited reduced declines in AIBL PACC scores with increasing sleep disturbances. Specifically, as sleep disturbances increased, decline in AIBL PACC scores was attenuated in rs12968026 minor allele homozygotes (CC) by 0.015 SD/year (*β *= 0.0148, SE = 0.0044, *p *= 0.0009), with a similar effect observed for rs3875089 minor allele homozygotes (CC) (*β *= 0.0140, SE = 0.0041, *p *= 0.0008). In both cases, no such relationships were seen with the alternative genotypes (*T+*: *β *= 0.0002, SE = 0.0007, *p *= 0.7378; *T+*: *β *= 0.0002, SE = 0.0007, *p *= 0.7435, respectively), reflecting significant differences between the slopes of the genotypes (Δ*β *= 0.0145, SE = 0.0045, *p *= 0.0013; Δ*β *= 0.0137, SE = 0.0042, *p *= 0.0012, respectively). All associations described remained significant after FDR correction, with no significant associations with Aβ burden surviving correction. For full results tables (including all dominant and recessive genetic models) see Table S8.

## 4 DISCUSSION

In CU older adults at increased risk for AD, due to Aβ accumulation, this study demonstrates that genetic variation in *AQP4* is associated with key AD‐related phenotypes, including cognition and brain structure, and that these relationships are moderated by sleep quality. By integrating genetic, neuroimaging, and self‐reported sleep data from the AIBL cohort, we extend prior findings and reveal novel gene–environment interactions that are associated with disease trajectory. These findings provide preliminary evidence that AQP4 variants may be linked to vulnerability or resilience in AD‐related phenotypes, with sleep potentially modifying these relationships, supporting further investigation of sleep as a modifiable factor in preclinical AD. However, longitudinal and interventional studies are needed to clarify directionality and clinical relevance.

The only direct SNP–phenotype association observed was for rs162007, where the minor allele (A) was associated with better cross‐sectional cognitive performance, specifically for AIBL PACC. rs162007 is an intronic variant that is also predicted to have regulatory function based on RegulomeDB (score 1f)^38^ and ForgeDB (score 10).^39^ Supporting its regulatory role, Genotype‐Tissue Expression (GTEx) data indicate that the minor allele is associated with reduced AQP4 expression in pituitary tissue.^40^ In addition, rs162007 is in complete linkage disequilibrium with rs162008, which has been shown to modulate *AQP4* expression in vitro using luciferase reporter assays, with the rs162008*T* (*corresponding to rs162007*A) allele linked to reduced gene expression.^41^ While these data support a potential regulatory role for this locus, the biological mechanisms underlying the observed association with cognition remain to be determined.

Building on this, interactions between *AQP4* variants and sleep disturbances were observed for cognitive outcomes. Individuals homozygous for the rs12968026 minor allele (C) exhibited attenuated cognitive decline in the context of increased sleep disturbances. This variant is predicted to have regulatory function,^38^, ^39^ with minor allele carriage associated with reduced *AQP4* expression in pituitary tissue.^40^ These findings differ from those in previous reports for rs72878776, which is in linkage disequilibrium with rs12968026, where minor allele carriers showed higher Aβ burden with shorter sleep duration.^13^ Similarly, homozygosity for the rs3875089 minor allele (C) was associated with reduced cognitive decline in the presence of increased sleep disturbances. This is consistent with prior studies reporting slower cognitive decline in AD^10^ and improved functional outcomes following traumatic brain injury (measured by the Glasgow Outcome Score)^42^ in carriers of this allele, although associations with increased risk of intracerebral hemorrhage have also been reported.^43^ Like rs12968026, rs3875089 is predicted to have regulatory function^38^, ^39^ and has been associated with reduced *AQP4* pituitary tissue expression.^40^ Together, these cognition findings suggest that the effects of *AQP4* variants may be context‐dependent, potentially interacting with sleep‐related processes.

Extending these findings, additional *AQP4* variants demonstrated interaction effects with sleep measures on brain structural outcomes. Individuals homozygous for the rs68006382 minor allele (GG) who reported longer sleep duration exhibited smaller white matter volumes. The rs68006382 minor allele has been linked to accelerated cognitive decline and faster progression to MCI in Parkinson's disease,^12^ supporting a potential role in neurodegeneration. Similarly, minor allele carriers of rs7240333 exhibited larger cross‐sectional ventricular volumes with longer sleep onset latency. Previous work reported a protective association of rs7240333 minor allele carriage in Parkinson's disease, including associations with better memory performance, although these findings did not survive correction for multiple testing.^12^

Notably, rs2339214 was the only variant associated with multiple outcomes, with consistent effects observed across brain structure measures. Minor allele (A) carriage was associated with less favorable outcomes in the context of poorer sleep, including greater rates of gray matter atrophy with shorter sleep duration and increased ventricular expansion with worsening global PSQI scores. A risk effect for this SNP was reported previously in the AIBL cohort, where minor allele homozygosity was associated with higher brain Aβ burden in the context of longer sleep duration.^13^ Together, these findings further support a role for rs2339214, with adverse effects emerging in the presence of disrupted sleep.

Finally, rs151245 was associated with gray matter atrophy in a genotype‐dependent manner, where among individuals homozygous for the major allele (TT), shorter sleep duration was associated with a faster rate of gray matter atrophy. No prior associations for this variant have been reported in the literature. rs151245 is an upstream variant predicted to have regulatory function (RegulomeDB score 1f; ForgeDB score 8),^38^, ^39^ suggesting a potential role in gene regulation, although its biological relevance in this context remains to be determined.

Importantly, although the magnitude of the volumetric and cognitive changes observed in this study is modest, the context of these effects suggest they reflect meaningful biological variation rather than noise or methodological artifact. Structural MRI measures demonstrate high test–retest reliability, indicating that the observed volumetric differences are unlikely to be attributable to measurement error.^44^, ^45^ In addition, the magnitude of these effects represents a modest proportion of expected age‐related brain volume change, placing them within a biologically plausible range.^46^ Similarly, for cognitive outcomes, where AIBL PACC scores are standardized to a cognitively healthy reference group within the cohort, differences observed between genotype groups reflect meaningful deviation from this baseline.

When interpreting the findings of this study, consideration should be given to the relationship between *AQP4* genetic variation, Aβ accumulation, and downstream phenotypes, including brain structure and cognition. It was hypothesized that genetic variation within *AQP4* may influence these downstream phenotypes due to the AQP4 proteins’ proposed role in facilitating Aβ clearance via interstitial fluid movement. However, in this study, many of the variants associated with brain volume and cognition differences were not significantly associated with Aβ burden, raising questions about the proposed underlying biological mechanisms. While Aβ‐independent pathways cannot be ruled out, it is also possible that the study design affected our ability to detect associations with Aβ burden. Specifically, participants were selected based on evidence of existing Aβ accumulation. As Aβ deposition is a gradual process that occurs over decades,^2^ any genetic associations with Aβ burden might have occurred earlier in the disease trajectory. As such, contemporaneous associations might not have been detectable, and instead, the downstream consequences, namely, atrophy and cognitive decline, were observed. Alternatively, the absence of associations between *AQP4* variants and Aβ burden suggests that the associations observed with cognition and brain volumes may reflect differences in other biological mechanisms. For example, AQP4 has been implicated in alterations to cerebral fluid dynamics,^47^ neuroinflammation,^7^ and astrocytic functioning,^6^ which could contribute to brain atrophy and cognition.

Several potential limitations should be considered when interpreting the results of this study. First, while cross‐sectional outcome variables were drawn from the same collection as the sleep characteristics data, longitudinal outcome variables were assessed in relation to baseline sleep values. It is acknowledged that sleep characteristics may change over time; however, in this study, paired *t*‐tests between sleep data collected at different time points showed no statistical difference from the initial time point, suggesting sleep characteristics in this subset of the AIBL cohort remained relatively stable over time. Additionally, this study relied on self‐reported sleep behavior assessed using the PSQI. While the PSQI has been shown to be a reliable, cost‐effective, and valid measure of sleep, there is the potential for data to be confounded by recall bias, subjective assessment, and a participant's recollection. Longitudinal analyses, particularly of brain volumetric outcomes, were conducted in a relatively modest sample size, which may have limited power to detect small to moderate genetic and interaction effects. While consistent associations were observed for some brain volume measures, these findings should be interpreted with appropriate caution and warrant replication in larger independent cohorts. In addition, the current study focused on investigating *AQP4* genetic variants in a cohort of individuals who were accumulating Aβ but were CU at baseline. Restriction of the cohort to Aβ accumulators may induce collider bias if *AQP4* variants and sleep jointly influence Aβ accumulation. Sensitivity analyses in unselected participants or broader populations are needed to establish whether the reported associations reflect true causal relationships or potential selection artifacts. Future studies may also expand on this to understand whether these associations vary across the disease continuum by examining other cohorts. Finally, the generalizability of these findings is limited by the demographic characteristics of the AIBL cohort, which is predominantly Caucasian and highly educated.

In conclusion, this study demonstrates that, in a cohort of CU older adults at risk for AD, genetic variation in *AQP4* is associated with differences in cognition and brain volume, as well as, to a lesser extent, brain Aβ burden. The findings here also provide evidence for gene–environment interactions, whereby the relationships of *AQP4* variants with AD‐related phenotypes are moderated by measures of sleep quality. If these associations reflect causal relationships, sleep interventions may mitigate genetic risk; however, randomized trials would be required to establish causality. Given that sleep is a modifiable behavior, these results may help identify individuals who could benefit most from targeted lifestyle interventions. While individual *AQP4* SNPs were associated with varying phenotypes, the overall findings support a broader role for *AQP4* genetic variation in AD. This is further supported by the fact that many of the implicated variants are predicted to have regulatory functions. As such, future studies should investigate the functional consequences of these variants to better understand the biological mechanisms underpinning these associations.

## CONFLICT OF INTEREST STATEMENT

Tenielle Porter, Ayeisha Milligan Armstrong, Eleanor K. O'Brien, Vincent Doré, Pierrick Bourgeat, Mitchell Turner, Belinda M. Brown, Stephanie R. Rainey‐Smith, and Simon M. Laws report no disclosures. Victor L. Villemagne is and has been a consultant or paid speaker at sponsored conference sessions for Eli Lilly, Life Molecular Imaging, ACE Barcelona, and IXICO. Paul Maruff is a full‐time employee of Cogstate Ltd. Christopher C. Rowe has served on scientific advisory boards for Bayer Pharma, Elan Corporation, GE Healthcare, and AstraZeneca; has received speaker honoraria from Bayer Pharma and GE Healthcare; and has received research support from Bayer Pharma, GE Healthcare, Piramal Lifesciences, and Avid Radiopharmaceuticals. Author disclosures are available in the Supporting Information.

## CONSENT STATEMENT

The AIBL study has been approved by the human research ethics committees at Austin Health, St Vincent's Health, Ramsay Health Care WA|SA, and Edith Cowan University. Participants provided written informed consent prior to undergoing study procedures.

## Supporting information



Supporting Information

Supporting Information

Supporting Information
