# Supplementary material for: Evidence for direct and sleep‐moderated relationships between aquaporin‐4 genetic variants and Alzheimer's disease phenotypes
Source: Alzheimers Dement. 2026 May 29;22(6):e71516. doi: 10.1002/alz.71516 (PMC13239059; doi:10.1002/alz.71516)
Supplement: Supplementary file 2 — Supporting Information [file ALZ-22-e71516-s002.docx]

**SUPPLEMENTARY MATERIAL**

**Supplementary Figures**


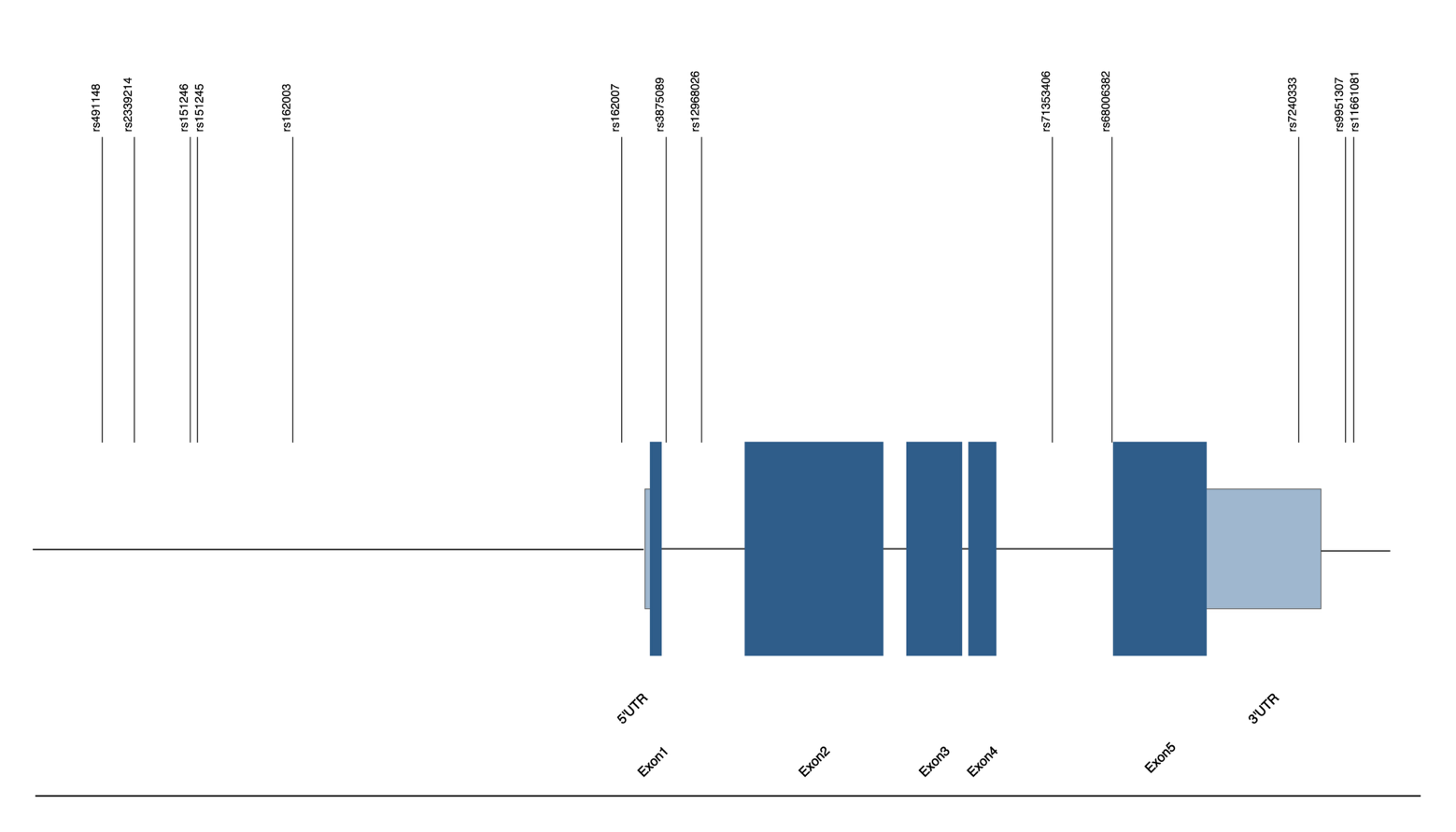


**Supplementary Figure 1. Schematic of *AQP4* gene**

A schematic diagram of the *AQP4* gene and the location of the variants assessed in this study. Abbreiations: UTR, untranslated region.
